# Supplementary material for: Comparative multiomics analysis of cell physiological state after culture in a basket bioreactor
Source: Sci Rep. 2022 Nov 23;12:20161. doi: 10.1038/s41598-022-24687-4 (PMC9686226; doi:10.1038/s41598-022-24687-4)
Supplement: Supplementary file 1 — Supplementary Information 1. [file 41598_2022_24687_MOESM1_ESM.zip › raw data/Proteomic raw data/6.KEGGenrichment/EG.vs.CG/UP/pathway/src/03013.html]

map03013


- K14295

- Up regulated proteins

A0A0D9QZ96(Description:RanBD1 domain-containing protein;Gene:NUP50;log2FC:1.2624)

- K12878

- Up regulated proteins

A0A0D9RYG7(Description:Death domain-containing protein;Gene:THOC1;log2FC:1.3665)

- K03259

- Up regulated proteins


A0A0D9R2B7(Description:Uncharacterized protein;Gene:EIF4E2;log2FC:1.8125)

- K03258

- Up regulated proteins

A0A0D9QZT6(Description:RRM domain-containing protein;Gene:EIF4B;log2FC:2.5466)

Close
